# La tuberculose cutanée: observation de six cas confirmés au CHU Souro SANOU (CHUSS) de Bobo-Dioulasso (Burkina Faso)

**DOI:** 10.11604/pamj.2013.16.50.3315

**Published:** 2013-10-11

**Authors:** Jean Baptiste Andonaba, Fatou Barro-Traoré, Téné Yaméogo, Boukary Diallo, Nina Korsaga-Somé, Adama Traoré

**Affiliations:** 1 Service de dermatologie, Centre Hospitalier Universitaire Souro Sanou, Bobo-Dioulasso, Burkina Faso; 2 Service de dermatologie, Centre Hospitalier Universitaire Yalgado Ouédraogo, Ouagadougou, Burkina Faso

**Keywords:** Tuberculose, peau, ganglion, Burkina Faso, Tuberculosis, skin, lymph node, Burkina Faso

## Abstract

La localisation cutanée de la maladie tuberculeuse demeure une forme rare et représente seulement 2,1% des localisations. L'objet de cette étude est de rapporter le profil épidémiologique, anatomoclinique et évolutif des cas de tuberculose ganglio-cutanée diagnostiqués dans un CHU au Burkina Faso. La fréquence de la tuberculose cutanée est très faible au CHUSS. Six cas ont été diagnostiqués entre 2004 et 2010, soit une fréquence de un cas par an. La durée d’évolution des cas allait de deux jusqu’à dix ans avant leur diagnostic. Les lésions observées étaient: trois scrofulodermes, trois gommes, une tuberculose testiculaire associée à un mal de Pott, un cas de polyadénopathies et des cicatrices atropho-rétractiles dans la plupart des cas. Sur le plan anatomopathologique, des granulomes tuberculoïdes ont été mis en évidence dans tous les cas avec une forte réaction tuberculinique à l'IDR. Sous antituberculeux pendant six mois, l’évolution a été bonne dans tous les cas mais au prix de séquelles cutanées cicatricielles inesthétiques. Son ampleur reste peut-être encore méconnue. Le renforcement du plateau technique du CHU et une bonne collaboration interdisciplinaire contribuerait à un meilleur diagnostic et prise en charge de cette affection.

## Introduction

Le *Mycobacterium tuberculosis* ou bacille de Koch (BK) est réputé pour sa prédilection à atteindre plusieurs organes. Sa localisation phare, à l'origine de la tuberculose pulmonaire, est un problème majeur de santé publique [1]. Certaines localisations extrapulmonaires, classiquement peu fréquentes, ont vu leur fréquence croître dans les pays où l'infection par le VIH est importante; c'est notamment le cas de la tuberculose pleurale [2]. La localisation cutanée de la maladie demeure une forme rare et représente seulement 2,1% des localisations [3]. Son diagnostic est difficile en raison du polymorphisme des tableaux anatomocliniques et de la multiplicité des diagnostics différentiels [4]. L'objet de cette étude est de rapporter le profil épidémiologique, anatomoclinique et évolutif des cas de tuberculose cutanée diagnostiqués dans un CHU au Burkina Faso.

## Patients and observations

### Cas 1

**Figure 1:**
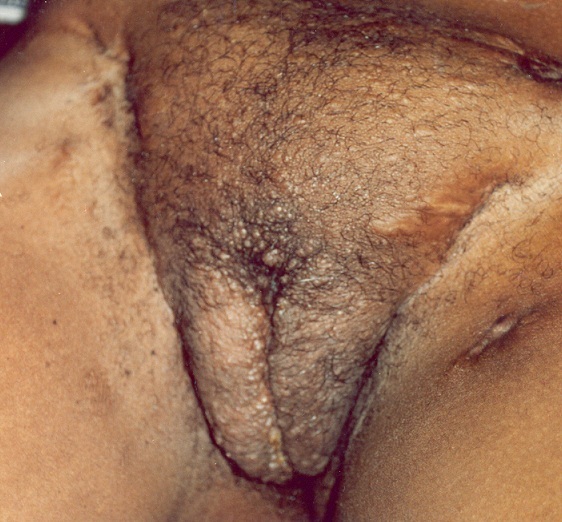
Esthiomène de la vulve et cicatrices rétractiles chez la patiente 1

**Figure 2:**
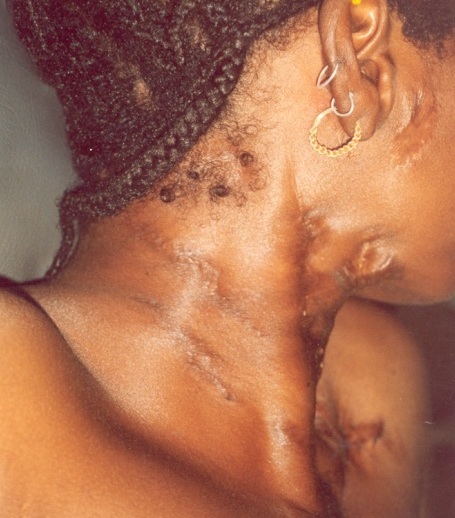
Cicatrices atropho-rétractiles chez la patiente 1

Madame D.D., âgée de 20 ans, femme au foyer, a consulté dans le service de dermatologie du CHUSS en 2004 pour des ulcérations cutanées, des cicatrices rétractiles et un œdème génital. Les antécédents notaient une cicatrice de BCG et une absence de contage et d'antécédent personnel de tuberculose. Le parcours thérapeutique a duré environ huit ans avec divers traitements anti syphilitique, anti chlamydia. A l'examen, l’état général était relativement conservé avec une notion d'amaigrissement non chiffré et une température à 37°C. La région cervicale, les membres supérieurs étaient le siège d’écrouelles et de cicatrices rétractiles. La cuisse gauche était le siège d'une gomme en voie de ramollissement. On notait un esthiomène de la vulve avec efflorescence de micronodules blanchâtres de tailles variables (Figure 1). Par ailleurs on a observé des adénopathies inguinales et cervicales infra-centimétriques. La sérologie pour le VIH, la sérologie syphilitique, la recherche mycologique et la recherche d'antigènes de chlamydiae étaient négatives. Les radiographies du squelette et du thorax et l’échographie abdominales étaient normales. La culture sur milieux de Lowenstein n'a pas pu être faite pour absence de réactifs et de milieux. L'intradermoréaction (IDR) à la tuberculine était fortement positive (15 mm) et l'histologie a confirmé le diagnostic de tuberculose cutanée en montrant un granulome tuberculoïde avec des cellules géantes (follicule de Koester caractéristique). Le traitement antituberculeux basé sur la quadrithérapie par isoniazide, rifampicine, éthambutol (E) et pyrazinamide (Z) pendant 2 mois, puis l'isoniazide (H) et la rifampicine (R) pendant 4 mois (2 ERH, 4RH), avec le suivi conjoint d'un pneumologue a permis une guérison avec séquelles à types d'esthiomène de la vulve, de lésions atropho-cicatricielles et des brides rétractiles (Figure 2).

### Cas 2

Madame K.S., 30 ans, femme au foyer, a été adressée dans le service de dermatologie en 2005 par un gynécologue pour des nodules et un ‘dème de la vulve. Les antécédents étaient sans particularité avec une notion d'amaigrissement non chiffré. Le parcours thérapeutique a été long (dix ans). Elle a reçu à deux reprises un traitement anti lépreux, des traitements anti syphilitiques et anti chlamydia sans succès. On notait une température à 37°C et un amaigrissement non chiffré. A l'examen on a retrouvé des cicatrices atrophiques et rétractiles sur les deux aires inguinales, un esthiomène de la vulve, une gomme ulcérée de la cuisse droite et une efflorescence de nodules de tailles variables sur la vulve avec un prurit important. On a également observé des micro-adénopathies inguinales. L'IDR à la tuberculine était fortement positive (16mm). L'histologie a confirmé le diagnostic. Les autres examens étaient sans particularité. La thérapie anti tuberculeuse (2 ERHZ, 4 RH), a permis une guérison avec des séquelles (esthiomène, cicatrices atrophiques et rétractiles). Le bénéfice fonctionnel du traitement était largement supérieur au bénéfice physique.

### Cas 3

**Figure 3:**
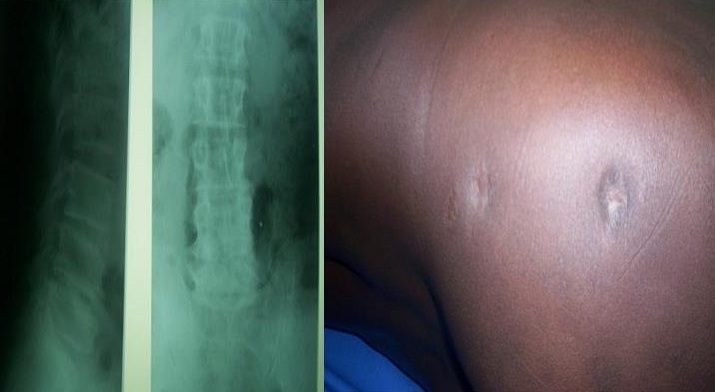
Tuberculose multifocale, cutanée, vaginale et osseuse (Patient 3)

Monsieur N.D., âgé de 48 ans, cultivateur, nous a été adressé de la chirurgie en 2005 pour un lymphœdème scrotal. Dans les antécédents on avait une notion de contage lointain sans cicatrices de BCG. A l'examen, on retrouvait un état général conservé avec une notion d'amaigrissement non chiffré, un esthiomène du testicule dont le diamètre atteignait 10 cm, suintant avec peau lisse, des cicatrices rétractiles sur les aires ganglionnaires inguinales et les fesses (Figure 3). Quelques adénopathies inguinales non inflammatoires étaient observées. Ces lésions évoluaient depuis six ans. La radiographie du rachis a objectivé un mal de Pott lombaire (Figure 3). L'IDR à la tuberculine était fortement positive (16mm). L'histologie a confirmé le diagnostic de la tuberculose. Les autres examens à la recherche d'autres localisations de la tuberculose, d'infection mycosique, chlamydienne et syphilitique étaient normaux. Sous la thérapie anti tuberculeuse (2 ERHZ, 4 RH), l’évolution a été favorable pour le mal de Pott et l'hydrocèle vaginale au prix de cicatrices atropho-rétractiles.

### Cas 4

**Figure 4:**
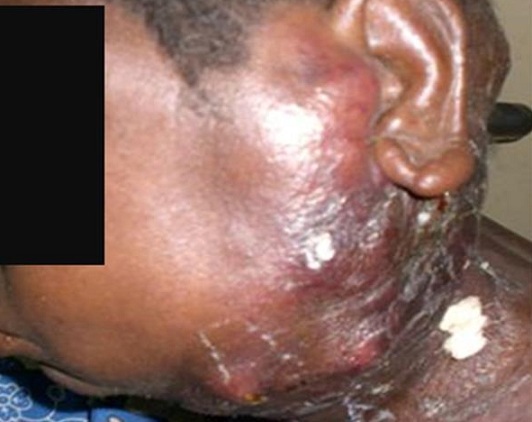
Adénites tuberculeuses suppurées chez la patiente 4

Mme A.K. âgée de 52 ans, femme au foyer a été hospitalisée en 2007 dans notre service pour polyadénites suppurées cervicales sous angulo-maxillaires et occipitales (Figure 4), évoluant depuis cinq ans dans un contexte fébrile (39°8). Elle avait été hospitalisée à plusieurs reprises en médecine interne, en chirurgie générale et chirurgie maxillo-faciale; Deux biopsies pratiquées n'ont pas été contributives. Sous traitements locaux et association d'antibiotiques synergiques il y'avait des périodes de rémission suivies de reprises évolutives. Dans les antécédents on retrouvait une notion de tuberculose dans l'entourage et une absence de Vaccination BCG. L'IDR à la tuberculine était fortement positive (18mm). L'histologie après une troisième biopsie a confirmé le diagnostic de la tuberculose. Les autres examens à la recherche d'autres localisations de la tuberculose et de pathologies associées étaient normaux. La thérapie anti tuberculeuse (2 ERHZ, 4 RH) a été efficace et la guérison a été obtenu avec des cicatrices atropho-rétractiles.

### Cas 5

**Figure 5:**
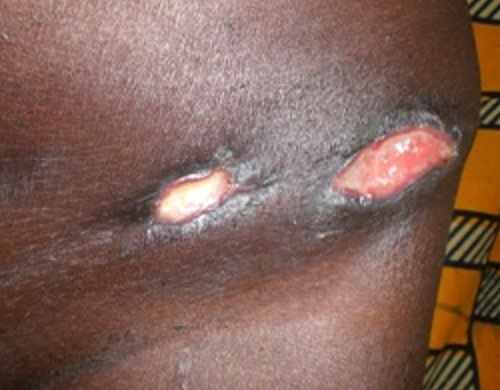
Scrofulodermes de l'aine chez la patiente 5 VIH1+

Mlle D.S, femme au foyer, âgée de 23 ans sous ARV pour une infection à HIV1 nous a été adressée par le service de gynécologie en 2008, pour des ulcérations chroniques et une altération de l’état général. Il n'y avait pas d'antécédents notables familiaux ou personnels chez cette patiente qui présentait à l'examen des ulcérations propres, indolores de tailles variables siégeant sur les régions inguinales (Figure 5), l'abdomen et le dos et des cicatrices rétractiles sur les aines; ces lésions évoluaient depuis environ deux ans avec des rémissions, des récidives. La patiente étaient amaigrie et fébrile (39°7C). On retrouvait de multiples adénopathies axillaires et inguinales infra-centimétriques. Le bilan radiologique n'a pas objectivé d'autres localisations. Les autres examens à la recherche d'autres localisations de la tuberculose et d'autres pathologies étaient normaux. Le taux de CD4 était bas 99/µl. L'IDR à la tuberculine était positive (14mm). L'histologie a confirmé le diagnostic au troisième prélèvement. Sous la thérapie anti tuberculeuse (2 ERHZ, 4 RH), l’évolution a été favorable aux prix de cicatrices atrophie-rétractiles inesthétiques.

### Cas 6

**Figure 6:**
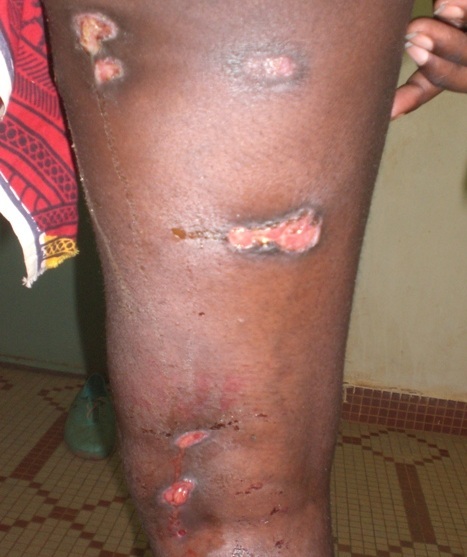
Gommes ulcérées de la cuisse chez la patiente 6 VIH1+

Mme B.Z., éleveur, âgée de 24 ans a consulté en 2010 dans le service de dermatologie pour de multiples ulcérations suintantes évoluant depuis deux ans environ. Dans les antécédents de la patiente, on retrouvait une notion de contage lointain, sans cicatrices de BCG ni de primo infection tuberculeuse patente à l'enfance. L’état général était conservé avec un amaigrissement non chiffré et une fièvre au long cours (38°5 au cours de l'examen). Il s'agissait de lésions nodulaires qui évoluaient vers le ramollissement, l'ulcération et l’écoulement d'un liquide séro-sanglant abondant (Figure 6). A l'examen, le flanc gauche, les deux bras et les deux cuisses étaient le siège de nombreuses lésions d’âges différents: gommes ramollies, ulcérations récentes avec écoulement, ulcérations en voie de cicatrisation, cicatrices atropho rétractiles. L'IDR à la tuberculine était positive (15mm). Le diagnostic a été confirmé par l'histologie. La sérologie VIH est revenue positive (VIH1+) avec un taux de CD4 à 200µl. Les autres examens à la recherche d'autres localisations de la tuberculose et de pathologies associées étaient normaux. La patiente a bénéficié de la thérapie anti tuberculeuse (2 ERHZ, 4 RH), et a été adressée à une structure spécialisée dans la prise en charge des PvVIH. L’évolution s'est faite vers la disparition des gommes et des ulcérations; il persiste cependant de larges cicatrices atropho rétractiles.

## Discussion

La fréquence de la tuberculose cutanée est très faible au CHUSS. La rareté de cette localisation du BK a été largement décrite [2–5]. Cependant, des fréquences plus importantes, de 3 à 29 cas par an ont été décrites en Inde, au Pakistan et à Sfax en Tunisie quoique cette affection représentait en général moins de 1% des consultations de dermatologie en ces lieux [3]. L'extrême faiblesse du nombre de cas observé au CHUSS de Bobo-Dioulasso pourrait être due à un sous-diagnostic des cas dans un contexte d'insuffisance du plateau technique. Le CHUSS compte deux dermatologues pour une population desservie de 1 469 604 habitants (Région des Hauts Bassins) et aucun anatomopathologiste. D’éventuels prélèvements biopsiques doivent être expédiés dans la capitale Ouagadougou située à 360 km pour l'examen anatomopathologique et aux frais de patients souvent démunis et sans couverture sociale (assurance-maladie). Ceci pourrait expliquer l'errance diagnostique et thérapeutique des patients, allant de deux jusqu’à dix ans (cas 2), avant que le diagnostic de tuberculose cutanée ne soit posé. Le caractère trainant de l'affection joint à l'absence d'atteinte d'organes mettant en jeu le pronostic vital chez nos patients pourrait également expliquer cette longue évolution avant le diagnostic. Les formes cliniques que nous avons observées sont comparables aux données de la littérature avec 4/6 cas de scrofuloderme (Figure 5). En effet, avec le lupus tuberculeux, le scrofuloderme constitue l'une des formes cliniques les plus fréquentes de la tuberculose cutanée dans sa forme multibacillaire [1, 6]. Les gommes tuberculeuses sont des lésions rares, prédominant aux membres inférieurs et survenant classiquement sur un terrain débilité, chez des enfants dénutris ou des patients immunodéprimés [2, 4]. Le cas 1 de notre série, une jeune femme de 20 ans, sans antécédent particulier, amaigrie, présentait un scrofuloderme et une gomme ulcérée de la cuisse. Si la recherche d'une infection au VIH s'est avérée négative chez cette patiente, la recherche d'une autre cause d'immunodépression devrait compléter les explorations. Le cas 6 qui présentait une tuberculose cutanée gommeuse étendue (Figure 6) avait une sérologie VIH positive. Le paradoxe vient du cas 5 qui était infecté par le VIH1 avec une immunodépression sévère et qui ne présentait que des scrofulodermes sans lésions gommeuses.

L'IDR positive chez tous les cas et l'image histologique de granulome tuberculoïde constitué d'un amas de cellules épithélioïdes et de cellules géantes de Langhans, entouré d'un manteau de cellules mononucléaires ainsi que l'altération de l’état général observés chez nos patients viennent compléter le tableau [6]. Deux biopsies faites en chirurgie n'ont pas été contributives (cas 4 et 5); en effet, ici comme ailleurs, les praticiens ne respectent pas toujours les normes en matière de biopsie cutanée en cas de tuberculose. Cette biopsie doit se faire en profondeur (jusqu’à l'hypoderme) pour optimiser les résultats histopathologiques [4].

Il faut cependant relever que le diagnostic bactériologique manque à ce tableau. Tigoulet rapporte toutefois la pauvreté des résultats bactériologiques des études de certaines grandes séries due d'une part à la difficulté de réalisation des cultures, d'autre part à la probable stérilité des prélèvements bactériologiques de certaines formes de tuberculose cutanée plutôt liée à une hyperréactivité immune [2]. Cette limite diagnostique ouvre ainsi un intérêt pour des nouvelles méthodes diagnostiques telles que la PCR.

Aucun enfant n'a été retrouvé dans notre série comme l'ont rapporté certains auteurs [3]. Depuis 2005, La couverture vaccinale du vaccin BCG est au-delà de 90% au Burkina Faso (OMS, statistiques par pays). La protection conférée par ce vaccin chez les enfants pourrait expliquer que des cas ne soient pas rencontrés [4].

Sur le plan thérapeutique, le traitement de la tuberculose cutanée repose sur les anti-bacillaires spécifiques du BK et il est identique au traitement de la tuberculose pulmonaire. Plusieurs antibiotiques sont associés pendant 3 à 12 mois selon des schémas thérapeutiques propres à chaque pays avec de bons résultats [2–6]. Dans notre série, l’évolution a été favorable dans tous les cas sous l'association en première phase de traitement d’éthambutol, de rifampicine, d'isoniazide et de pyrazinamide pendant deux mois, puis en deuxième phase, de rifampicine et d'isoniazide pendant quatre mois. Il faut toutefois souligner les nombreux traitements antérieurs reçus par les patients (anti chlamydia, anti syphilis, anti lépreux et diverses antibiothérapies non spécifiques); ils dénotent du spectre de diagnostics différentiels avec lesquels la tuberculose cutanée peut être confondue (syphilis, chlamydiase, lèpre, pyodermites). D'autre part, les séquelles cutanées cicatricielles à type d'esthiomène, observées chez deux de nos patients sont potentiellement le lit de phénomènes inflammatoires chroniques qui pourraient induire des fibroses et des blocages lymphatiques responsables de lésions pseudoéléphantiasiques (Figure 1) qui n'ont pas été retrouvées dans la littérature.

## Conclusion

La tuberculose cutanée, semble rare au CHU de Bobo-Dioulasso. Son ampleur reste peut-être encore méconnue. Ces cas montrent l'importance de la formation continue des agents de santé et le renforcement des moyens d'investigation pour un diagnostic précoce et une collaboration multidisciplinaire pour une meilleure prise en charge des patients.

## References

[1] OMS - Global tuberculosis control WHO report 2008.

[2] Tigoulet F, Fournier V, Caumes E (2003). Clinical forms of the cutaneous tuberculosis. Bull Soc Pathol Exot..

[3] Fenniche S, Ben Jennet S, Marrak H (2003). Cutaneous tuberculosis: anatomoclinical features and clinical course (26 cases). Ann Dermatol Venereol..

[4] Morand JJ, Cuguilliere A, Sayag J (1999). Tuberculose cutanée. Encycl Méd Chir - Dermatologie.

[5] Hassam B, Senoussi K, Bennouna-Biaz K, Lazrak B (1991). Profil épidémiologique des tuberculoses cutanées colligées au service de dermatologie d'Avicenne (1985 – 1990). Médecine du Maghreb..

[6] Khallafi S, Soualhi M, Zahraoui R (2006). La tuberculose cutanée: à propos de 23 cas. Revue des Maladies Respiratoires.

